# Modeling human neurodevelopmental disorders in the *Xenopus* tadpole: from mechanisms to therapeutic targets

**DOI:** 10.1242/dmm.012138

**Published:** 2013-08-07

**Authors:** Kara G. Pratt, Arseny S. Khakhalin

**Affiliations:** 1 University of Wyoming, 1000 E University Avenue, Laramie, WY 82071, USA; 2 Brown University, 45 Prospect Street, Providence, RI 02912, USA

## Abstract

The *Xenopus* tadpole model offers many advantages for studying the molecular, cellular and network mechanisms underlying neurodevelopmental disorders. Essentially every stage of normal neural circuit development, from axon outgrowth and guidance to activity-dependent homeostasis and refinement, has been studied in the frog tadpole, making it an ideal model to determine what happens when any of these stages are compromised. Recently, the tadpole model has been used to explore the mechanisms of epilepsy and autism, and there is mounting evidence to suggest that diseases of the nervous system involve deficits in the most fundamental aspects of nervous system function and development. In this Review, we provide an update on how tadpole models are being used to study three distinct types of neurodevelopmental disorders: diseases caused by exposure to environmental toxicants, epilepsy and seizure disorders, and autism.

## Introduction

Neurons have the amazing ability to self-assemble into highly organized circuits. These circuits give rise to our perceptions, thoughts and emotions, and determine how we experience our world. Disorders in neural development, therefore, can often compromise the quality of life. To date, there are no cures for prevalent neurodevelopmental disorders such as autism, epilepsy and schizophrenia, and there are many gaps in what is known about the underlying causes of these conditions. Animal models that allow for a disorder to be studied at multiple levels, from molecules to behavior, can provide a more complete understanding of the associated gene locus and underlying mechanism(s), thereby promoting the design of novel approaches for treatment and prevention.

**Fig. 1.**
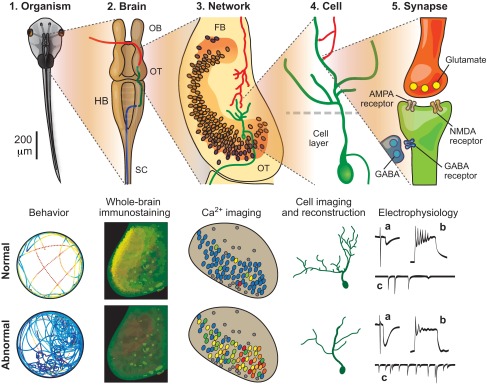
**The *Xenopus* tadpole as a research model, shown with key experimental techniques that are used to differentiate between normal and abnormal patterns of neural development.** (1) Top: view of the animal at ca. 3 weeks post-fertilization. Several behavioral tests can be used to assess brain development: for example, wild-type animals usually swim along the sides of the container (represented by a circle; bottom), whereas animals with altered excitation/inhibition balance tend to circle in the middle of it. (2) Top: general view of the brain. OB, olfactory bulbs; OT, optic tectum; HB, hindbrain: SC, spinal cord; red, projections from the retina; green, tectal projections to the hindbrain; blue, descending projections to the spinal cord. An isolated brain provides an accessible *in vitro* preparation, and whole-brain immunostaining (bottom) can be used to quantify global alterations in brain biochemistry (an exaggerated staining for GABA is shown). (3) Top: horizontal section of the optic tectum (OT) and caudal forebrain (FB); at this level, Ca^2+^ imaging can be used to detect abnormal seizure-like patterns of activity (bottom). (4) At the neuron level, *in vivo* or *ex vivo* imaging allows assessment of cell morphology development. (5) At the synaptic level, electrophysiology offers a way to quantify maturation of synaptic and intrinsic properties of the cell through recordings of (a) evoked synaptic responses, (b) spiking in response to current injections and (c) spontaneous synaptic activity. The figure is inspired by experimental data published in the following papers: (Aizenman et al., 2002; Bestman et al., 2006; Ruthazer et al., 2006; Pratt and Aizenman, 2007; Hewapathirane et al., 2008; Bollmann and Engert, 2009; Hiramoto and Cline, 2009; Straka, 2010; Bell et al., 2011; Marshak et al., 2012; Miraucourt et al., 2012).

The *Xenopus laevis* tadpole possesses many qualities that make it a powerful model to study disorders of the developing nervous system. First and foremost, essentially every stage of normal neural development, from neurogenesis and differentiation to axon pathfinding, synapse maturation and circuit refinement, has been studied in detail in *Xenopus* tadpoles (Cline and Kelly, 2012; Sanes et al., 2012). Such a detailed understanding of normal developmental processes is invaluable when seeking to determine how they can malfunction. Compared with mammalian neural circuits, those of the tadpole are simpler, yet homologous in their basic organization. For example, in the tadpole retinotectal circuit (Fig. 1), retinal ganglion cells (RGCs) in the eye project their axons to the brain, where they synapse onto tectal neurons in the contralateral optic tectum (Gaze, 1958; Sperry, 1963), a midbrain structure that is homologous to the mammalian superior colliculus. The RGC axons form a highly organized topographic map within their target structure, with neighboring RGCs making synapses onto neighboring tectal neurons. This mirrors what is observed in many mammalian sensory circuits, including those within the human nervous system. Furthermore, as in most mammalian excitatory synapses, RGC axons release glutamate, and tectal neurons express AMPA and NMDA glutamate receptors (Wu et al., 1996). Building on the contributions of the *Xenopus* model to the field of embryology, many aspects of *Xenopus* neural circuit development have been carefully studied, and meticulously described across the key developmental stages. For instance, it is well established that the axons of the RGCs reach the tectum at around developmental stage 39 [4–5 days post-fertilization (dpf)] (Holt, 1989; Dingwell et al., 2000), that the most dynamic phase of circuit formation – both morphologically and functionally – occurs between stage 44 and 47 (7–10 dpf), and that by stage 49 (∼16–24 dpf) the circuit becomes more refined and stable (Sakaguchi et al., 1984; Cline et al., 1996b; Pratt and Aizenman, 2007).

Experimentally, *Xenopus* tadpoles pose many advantages. Because of their transparency, and the extreme dorsal location of the brain, the axons and dendrites of single living neurons can be imaged *in vivo* (Harris et al., 1987; Ruthazer et al., 2003; Cohen-Cory, 2007; Li et al., 2011; Dong and Aizenman, 2012) and, better yet, in awake animals (Chen et al., 2012; Hossain et al., 2012). Time-lapse imaging of radial glia in the tectum has provided the first *in vivo* description of how neural activity affects the structure and function of these cells (Tremblay et al., 2009), and advances in morphometric software (Liu et al., 2009; Chen et al., 2012) have allowed for an improved ability to track and measure all processes in 3D across short intervals over long periods of time (Hossain et al., 2012). Furthermore, calcium imaging in tadpoles can be carried out readily (Tao et al., 2001; Junek et al., 2010; Xu et al., 2011), including *in vivo* recordings from intact awake animals (Chen et al., 2012; Podgorski et al., 2012; Imaizumi et al., 2013). Expression of genes of interest can also be achieved *in vivo*, using electroporation-based protocols (Haas et al., 2001; Haas et al., 2002; Bestman et al., 2006), or by mRNA injection into appropriate cells of the early embryo (Demarque and Spitzer, 2010). Because of the relatively high permeability of the tadpole blood-brain barrier, pharmacological manipulations of the nervous system are usually achieved by simply adding the pharmacological agent to the tadpole rearing solution. Electrophysiological techniques have been successfully employed in *Xenopus* to quantify network connectivity (Pratt and Aizenman, 2007; Li et al., 2009; Pratt and Aizenman, 2009; Straka and Simmers, 2012), synaptic maturation (Wu et al., 1996; Akerman and Cline, 2006; Aizenman and Cline, 2007; Deeg et al., 2009; Khakhalin and Aizenman, 2012), synaptic plasticity (Engert et al., 2002; Mu and Poo, 2006; Pratt et al., 2008; Tsui et al., 2010) and cell intrinsic properties (Aizenman et al., 2003; Pratt and Aizenman, 2007; Winlove and Roberts, 2011). The behaviors controlled by corresponding neural circuits, including several types of escape behaviors (Roberts et al., 2000; Wassersug and Yamashita, 2002; Dong et al., 2009; Sillar and Robertson, 2009), orienting reflexes (Pronych et al., 1996; Simmons et al., 2004; Straka, 2010) and social behaviors (Katz et al., 1981; Villinger and Waldman, 2012), have been well described, and can be experimentally manipulated (Lum et al., 1982; Jamieson and Roberts, 2000; Wassersug and Yamashita, 2002; Simmons et al., 2004; Dong et al., 2009; Straka, 2010). To sum up, these experimental approaches enable developing neural circuits to be examined at the molecular, cellular and behavioral levels – all in the same organism (Fig. 1). Moreover, humans are genetically closer to *Xenopus* than to similar model organisms, such as zebrafish, because teleosts (the ray-finned fishes) are known to have divergent and highly specialized genomes (Postlethwait et al., 2004; Nakatani et al., 2007; Rash et al., 2012). Combined with the relatively low cost of housing, large number of embryos generated from one mating, and the ease of embryologic (De Robertis, 2006; Harland and Grainger, 2011; Pai et al., 2012) and surgical (Constantine-Paton and Capranica, 1976; Filoni, 2009; McKeown et al., 2013; Elliott et al., 2013) manipulations, these qualities render *Xenopus* an ideal model for neurodevelopmental research.

In this Review, we first highlight how tadpoles have been used as a model for assaying the effects of environmental chemicals on neurodevelopment, and how the model itself has evolved and been refined over the years. We then describe a recently designed tadpole model of epileptic seizures that has already led to the finding of a built-in protective mechanism that is activated in response to a seizure. Finally, we present a new and exciting tadpole model to study autism.

## Characterizing the effects of environmental toxicants on development in a tadpole model

During development, neural circuits can be particularly sensitive to chemicals in the environment. In humans, for example, doses of methylmercury that are neurotoxic to the embryonic central nervous system (CNS) have no effect on the maternal CNS (Castoldi et al., 2001). Similarly, exposure of the developing nervous system of a rat pup to lead (a heavy metal) often results in encephalopathy, whereas the mature rat brain remains unaffected when exposed to the same amount of lead (Holtzman and Hsu, 1976). Thus, chemicals in the environment that are deemed to be innocuous to the adult CNS can be harmful to a developing brain.

Having been used for decades by researchers in academia as well as the US government’s Environmental Protection Agency (EPA) to assay toxic and teratogenic effects of environmental chemicals, the *Xenopus* tadpole is not new to the field of embryotoxicology (Dumpert and Zietz, 1984; Degitz et al., 2003; Richards and Cole, 2006; Berg et al., 2009). The tadpole has served as a workhorse for these studies mostly because their metamorphosis from tadpole to frog depends entirely on thyroid hormone (TH) (Damjanovski et al., 2000), and, in turn, one of the most prevalent environmental contaminants are the TH inhibitors, a major class of endocrine disruptors. In the tadpole, if TH action is inhibited, metamorphosis stalls, whereas exposure to TH in pre-metamorphic tadpoles induces precocious metamorphosis (Helbing et al., 2007). Because the progression of metamorphosis is well described and obvious, alterations can be readily identified. Hence, this became a convenient way to test many classes of chemicals for their ability to disrupt TH activity (Gutleb et al., 2000; Tietge et al., 2005; Cheng et al., 2011; Lorenz et al., 2011). One of the major targets of TH, however, is the brain, where disruption of normal TH activity can lead to neurodevelopmental defects (Zoeller and Crofton, 2000). Given that it is unlikely that the more subtle defects associated with neurodevelopment (such as incomplete synapse refinement for example) would disrupt the relatively gross changes associated with metamorphosis (such as loss of the tail, emergence of limb buds and formation of lungs), disorders in nervous system development could go undetected. Thus, it was necessary to develop a more sensitive molecular approach for the identification of neural-specific molecular markers that are associated with TH disruption. Experiments using quantitative reverse transcriptase PCR (qRT-PCR) revealed that the TH inhibitors methimazole and perchlorate alter neural TH receptor expression in brain tissue of stage-54 tadpoles (Zhang et al., 2006). Furthermore, in a detailed study combining cDNA array analysis and qRT-PCR, perchlorate was found to significantly increase the expression of several neural mRNAs (Helbing et al., 2007), including the mRNA for β-amyloid precursor protein, a protein whose improper processing has been highly implicated in Alzheimer’s disease, and mRNAs that encode for myelin basic protein and myelin proteolipid protein, both of which are major components of the myelin sheath that insulates axons and facilitates appropriate action potential conduction. The effects that these perchlorate-induced increases in mRNAs could have on the developing tadpole brain, however, remain unknown.

Advances in both imaging and electrophysiological approaches have enabled the tadpole to become a powerful *in vivo* model for investigating, at a high resolution, how environmental chemicals can affect developing neurons. For instance, a study using the *Rana pipiens* tadpole has shown that chronically exposing tectal neurons to low, sub-micromolar levels of lead decreases both RGC axon arbor area and branchtip number. The same group showed that acute lead exposure weakens synaptic transmission between RGC axons and tectal dendrites (Cline et al., 1996a). More recently, the *Xenopus* tadpole was used to characterize the effects of sub-lethal concentrations of methylisothiazolinone (MIT; a biocide commonly used in several cosmetics, including shampoo) on many aspects of nervous system function (Spawn and Aizenman, 2012). For example, overall visual system function was tested using protocols designed to characterize tectum-dependent and thalamus-dependent visual behaviors (Dong et al., 2009). MIT-exposed tadpoles displayed deficits in only the tectum-dependent visual behavior, suggesting a malfunction in retinotectal synaptic transmission. Although no differences were observed in synaptic transmission between RGC inputs and tectal neurons in the MIT-treated tadpoles, the pattern of the recurrent tecto-tectal connectivity – which is activated by RGC inputs – (Fig. 1C) was altered in a way that suggests lack of circuit refinement. At the single-neuron level, no differences in intrinsic excitability and synaptic strengths were observed in MIT-treated tadpoles (Fig. 1D). In summary, the deficits in tectum-dependent visual behavior and unrefined tecto-tectal connectivity, combined with the absence of noticeable changes in intrinsic or synaptic properties, suggest that chronic MIT exposure causes problems at the circuit level and not at the single-cell level. For neurotoxicology research, this study exemplifies how chronic exposure to concentrations of a chemical with no noticeable effects on either survival or morphology can still compromise a developing neural circuit. Overall, this study demonstrates the level of detail at which neurons and neural circuits can be assayed using the *Xenopus* tadpole, and, more specifically, how a deficit in a behavior can be tracked down and studied at the circuit and single-neuron level.

## A tadpole model for epileptic seizure

In addition to environmental toxins, developing circuits are particularly susceptible to epileptic activity. A protocol to reliably induce controlled seizures in the *Xenopus* tadpole has been developed (Hewapathirane et al., 2008) and has already led to a fundamental insight into how endogenous polyamines can play a neuroprotective role in response to an epileptic seizure (Bell et al., 2011).

The development of the tadpole model for studying epileptic seizures began with a detailed characterization of the ability of several different classes of chemoconvulsants to reliably induce seizures in stage-47 tadpoles. Several different classes of known convulsants were tested: GABA-receptor antagonists [pentylenetetrazole (PTZ), picrotoxin and bicuculline], glutamate receptor agonists (kainate), muscarinic receptor agonists (pilocarpine) and potassium channel inhibitors (4-aminopyridine) (Hewapathirane et al., 2008). All of these convulsants seemed to produce a common type of behavioral seizure in tadpoles. The behavior commences with intermittent bouts of rapid swimming, followed by immobility, deviations from the normal head-down tail-up posture, and lateral movements of the head, followed ultimately by full-blown seizure behavior – C-shaped contractions evoked by abnormal unilateral axial muscle contractions that are so strong that they result in the entire tadpole displaying a stereotypical ‘C’ shape. Because all of the different classes of convulsant induced the same type of seizure, it was concluded that this is a ‘true’ seizure rather than the effects of a particular drug on motor function. The GABA receptor antagonist PTZ was determined to be the optimal chemoconvulsant for the tadpole seizure model because it reliably induces the stereotypical C-shape contractions at doses that are neither lethal nor toxic, and *in vivo* field potential recordings in the optic tectum revealed robust epileptiform activity, i.e. high-amplitude spiking, in response to PTZ application. This epileptiform activity can be blocked completely by administration of the anti-epileptic drug valproate. An advantage of this model is that immobilization of the tadpole for electrophysiology or imaging experiments can be achieved using reversible paralytics or agar immersion, thereby allowing seizures to be studied in the absence of anesthetic agents (Hewapathirane et al., 2008).

In a recent study by Bell et al. (Bell et al., 2011) involving a protocol consisting of two consecutive PTZ-induced seizures and a combination of behavioral, electrophysiological and pharmacological experiments, it was shown that the first initial PTZ-induced seizure in a tadpole increases the production of polyamines [an observation that had also been reported in a rodent seizure model (Hayashi et al., 1993)]. Elevated polyamine levels were found to boost the production and release of the inhibitory transmitter GABA, which rendered tadpoles less prone to future seizures. Similarly, exposing tadpoles to enhanced visual stimulation led to increased GABA levels in the tectum, providing another compelling example of how GABA can function in a homeostatic manner in response to abnormally high levels of circuit activity (Miraucourt et al., 2012).

## Tadpole models for the study of autism spectrum disorders

Autism spectrum disorders (ASD) are paradoxical: the syndromes within this group present with a highly recognizable set of symptoms, yet they can be caused by a diverse array of genetic abnormalities and environmental insults, such as prenatal infection, hormonal exposure and teratogens (Newschaffer et al., 2007; Abrahams and Geschwind, 2008). Mutations in more than 40 genes have been shown to increase susceptibility to ASD, yet none of these mutations are completely penetrant, i.e. cause ASD with 100% probability (Lichtenstein et al., 2010; Neale et al., 2012). Furthermore, although the defining symptoms of ASD, such as deficits in language, social interactions and personal interests are manifested at the highest cognitive levels, the etiology of ASD has been linked to abnormalities in surprisingly fundamental aspects of nervous system functioning and development. This includes defects in synaptic plasticity (Krey and Dolmetsch, 2007; Markram et al., 2008; Bhakar et al., 2012), inhibition/excitation balance (Perry et al., 2007; Markram and Markram, 2010; Marín, 2012), microcircuitry organization (Geschwind, 2009) and neuron-glia interactions (Abrahams and Geschwind, 2008), as well as long-range underconnectivity and local overconnectivity, as a consequence of altered axon guidance and dendritic arborization (Rinaldi et al., 2008; Geschwind, 2009). These features suggest that ASD represents a uniquely human response to a broad class of developmental dysregulations (Peça and Feng, 2012) and, therefore, the mechanisms of ASD are likely to be successfully addressed in animal models not necessarily capable of expressing most behavioral and cognitive symptoms of ASD (Patterson, 2011). These animal models would include mammals, but also fish (Tropepe and Sive, 2003; Kabashi et al., 2011), birds (Panaitof, 2012), insects (Gatto and Broadie, 2011) and amphibians.

With this in mind, a successful experimental approach in *Xenopus* would entail looking directly at the changes caused by known ASD-associated developmental perturbations at the cellular and network levels. One of the unique benefits of the tadpole is the ease at which gene expression can be altered in individual neurons, and the convenience of registering the consequences of these perturbations *in vivo*, allowing a way to differentiate between cell-autonomous and network-level effects of ASD-associated mutations. As a good example, when a wild-type human *MeCP2* gene [a mutation in this gene causes Rett syndrome in humans, and is strongly comorbid with ASD (Samaco and Neul, 2011)] was overexpressed in *Xenopus* tectal neurons *in vivo*, these neurons were found to develop fewer, albeit longer, dendrites compared with normal tectal cells (Marshak et al., 2012). Hence, in *Xenopus*, as in humans and rodents, variations in *MeCP2* activity cause redistribution between close- and long-range network connections, which is one of the landmark circuit abnormalities in ASD (Geschwind, 2009). This work also illustrates that key transcription regulators are sufficiently conserved between *Xenopus* and humans (Amir et al., 1999), allowing the human *MeCP2* gene to interact (Marshak et al., 2012) with native *Xenopus* pathways (Stancheva et al., 2003).

Although not every gene linked to ASD in humans (Abrahams and Geschwind, 2008; Neale et al., 2012) has been identified and studied in *Xenopus* thus far (Hellsten et al., 2010), a large proportion have been; see Table 1 for a list. Among all ASD-related genetic conditions, the one that is most researched in a *Xenopus* model is Fragile X syndrome, which is linked to the deactivation of a single gene, *FMR1* (Abrahams and Geschwind, 2008; Levenga et al., 2010; Bhakar et al., 2012). The tadpole can be easily employed for studying associated network pathology via single-gene manipulation in the developing embryo. It has already been shown that manipulation of the *fmr1* gene in the developing *Xenopus* embryo disrupts proper somite formation (Huot et al., 2012), and it will be interesting to observe how altered *FMR1* expression affects developing neural circuits in humans. The Fragile-X-related genes are well described in *Xenopus* (both *X. tropicalis* and *X. laevis*), with frogs having fewer homologs within the gene family (*fmrp* and *fxr1p*) (Lim et al., 2005) than do humans (*FMRP*, *FXR1P* and *FXR2P*), as well as fewer isoforms per gene (Huot et al., 2005). Even so, it has been demonstrated that antibodies to human FMRP and FXRP proteins are effective against *Xenopus* proteins (Blonden et al., 2005; Huot et al., 2005), and that expression of human *FMRP* or *FXRP* rescues *fmrp* and *fxr1p* knockdown phenotypes in *Xenopus*, respectively (Huot et al., 2005; Gessert et al., 2010).

**Table 1.**
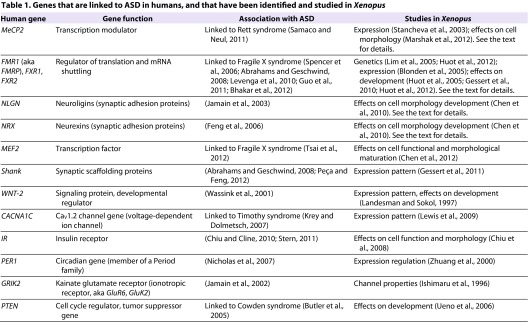
Genes that are linked to ASD in humans, and that have been identified and studied in *Xenopus*

Neuroligins and neurexins (synaptic adhesion proteins), together with Shank and PSD-95 scaffolding proteins, make up another functional group of proteins that share high degrees of homology between *Xenopus* and mammals (Ichtchenko et al., 1996; Chen et al., 2010; Gessert et al., 2011), have mutations in corresponding genes linked to ASD in human patients (Jamain et al., 2003; Feng et al., 2006; Marín, 2012), and have been successfully studied in *Xenopus*. When neuroligin-1 function was disrupted in *Xenopus* tectal neurons by either overexpression of mutant forms of mouse *Nlg-1*, competitive saturation of native Nlg-1 extracellular domains with soluble neurexin motifs, or a knock-down of native *Nlg-1* with morpholinos, the dendritic tree maturation profile for these neurons was altered. Specifically, the tectal neurons failed to establish new synapses with RGC axons, demonstrated higher motility of dendritic filopodia, and had a simplified and immature dendritic arbor morphology overall (Chen et al., 2010). Moreover, by comparing effects of these perturbations on the dynamics of dendritic arbor development, the authors managed to convincingly reconstruct the pattern of protein interactions occurring during filopodia stabilization and synapse formation. This work provides another example of how *in situ* time-lapse imaging in a live tadpole brain (Bestman et al., 2012; Hossain et al., 2012) can be combined with genetic manipulations (Bestman et al., 2006; Bestman and Cline, 2008; Liu and Haas, 2011) and electrophysiology (Pratt and Aizenman, 2007) to dissect the functional role of target synaptic proteins and their influence on network formation (Chen and Haas, 2011).

Finally, *Xenopus* modeling can help us to probe the link between ASD and immune activation in the brain (Deverman and Patterson, 2009). Although increases in glia activation and levels of pro-inflammatory cytokines have been observed in individuals with ASD, it is unclear whether these phenomena contribute to the cause, or are a consequence of the disorder (Cohly and Panja, 2005). When tadpoles were chronically exposed to interleukins (IL-1β, IL-6) or tumor necrosis factor (TNFα), tectal neurons were overconnected (based on the electrophysiological evidence), synapses seemed more mature (based on the AMPA:NMDA ratio), and animals had abnormal sensory processing, and were susceptible to seizures (Lee et al., 2010), confirming that immune activation alone can trigger some components of the ASD phenotype.

## Conclusions

Overall, these experiments underscore the power of the tadpole model in addressing a question from the molecular to the systems level (Fig. 1). As discussed in the Introduction, there are many factors that make the *Xenopus laevis* tadpole a particularly well-suited model for studying neurodevelopmental disorders. The only major weakness of the *X. laevis* model is that, because this species is tetraploid, meaning that they carry four copies of each gene, genetic manipulations are relatively difficult compared with manipulations of diploid genomes. Recent advances in transgenic techniques have made it possible to generate transgenic *X. laevis* tadpoles (Kroll and Amaya, 1996). These transgenic tadpoles have been used with much success (Kroll and Amaya, 1996; Marshak et al., 2007; Takagi et al., 2013), a relevant example being the generation of tadpoles that expressed dominant-negative TrkB (BDNF/neurotrophin receptor) exclusively in retinal ganglion cells (Marshak et al., 2007). Still, there are issues that remain: with four copies of any given gene, it is virtually impossible to completely knock out its expression. Instead, researchers have resorted to expressing dominant-negative mutations (Hawley et al., 1995; Coen et al., 2007) that effectively inhibit the endogenous function of a gene, but are often non-specific, inhibiting more than a single gene product. In addition to the expression of dominant-negative mutations, RNA interference (RNAi) (Miskevich et al., 2006) and morpholinos (Root et al., 2008; Sharma and Cline, 2010) have been used successfully in *Xenopus* to knock down gene expression. What could prove to be an even better vertebrate model, especially for genetics, is the only diploid species of *Xenopus*, *Xenopus tropicalis* (Amaya et al., 1998). Being diploid renders the *X. tropicalis* more amenable to genetic manipulations. Other than the difference in the number of copies of genes, these two species are quite similar. Thus, as a vertebrate model, *X. tropicalis* offers all the advantages of the *X. laevis* model without the experimental complications that are inherent to a polyploid genome. Hence, the *X. tropicalis* tadpole has been predicted to be the animal model of the future (Amaya et al., 1998).

In conclusion, the *Xenopus* tadpole has been and continues to be a powerful and prolific model to study, at many levels, both normal and abnormal neural development. The ability to study developing neural circuits at these different levels and with high resolution allows the tadpole to contribute greatly to the identification of currently unknown targets to treat neurodevelopmental disorders in humans.
